# Decreased Prevalence of Lymphatic Filariasis among Diabetic Subjects Associated with a Diminished Pro-Inflammatory Cytokine Response (CURES 83)

**DOI:** 10.1371/journal.pntd.0000707

**Published:** 2010-06-15

**Authors:** Vivekanandhan Aravindhan, Viswanathan Mohan, Jayagopi Surendar, Maradana Muralidhara Rao, Nathella Pavankumar, Mohan Deepa, Ramanujam Rajagopalan, Vasanthapuram Kumaraswami, Thomas B. Nutman, Subash Babu

**Affiliations:** 1 Madras Diabetes Research Foundation, Chennai, India; 2 Dr. Mohan's Diabetes Specialties Centre, Chennai, India; 3 National Institutes of Health-International Center for Excellence in Research, Chennai, India; 4 Tuberculosis Research Center, Chennai, India; 5 Laboratory of Parasitic Diseases, National Institutes of Allergy and Infectious Diseases, National Institutes of Health, Bethesda, Maryland, United States of America; 6 SAIC-Frederick, Inc., NCI-Frederick, Frederick, Maryland, United States of America; University of Washington, United States of America

## Abstract

Epidemiological studies have shown an inverse correlation between the incidence of lymphatic filariasis (LF) and the incidence of allergies and autoimmunity. However, the interrelationship between LF and type-2 diabetes is not known and hence, a cross sectional study to assess the baseline prevalence and the correlates of sero-positivity of LF among diabetic subjects was carried out (n = 1416) as part of the CURES study. There was a significant decrease in the prevalence of LF among diabetic subjects (both newly diagnosed [5.7%] and those under treatment [4.3%]) compared to pre-diabetic subjects [9.1%] (p = 0.0095) and non-diabetic subjects [10.4%] (p = 0.0463). A significant decrease in filarial antigen load (p = 0.04) was also seen among diabetic subjects. Serum cytokine levels of the pro-inflammatory cytokines—IL-6 and GM-CSF—were significantly lower in diabetic subjects who were LF positive, compared to those who were LF negative. There were, however, no significant differences in the levels of anti-inflammatory cytokines—IL-10, IL-13 and TGF-β—between the two groups. Although a direct causal link has yet to be shown, there appears to be a striking inverse relationship between the prevalence of LF and diabetes, which is reflected by a diminished pro-inflammatory cytokine response in Asian Indians with diabetes and concomitant LF.

## Author Summary

Childhood helminth infections can reduce the risk and severity of allergies and autoimmune diseases, by means of immunomodulation, and a decrease in helminth infections could potentially account for the increased prevalence of these diseases in the western world (hygiene hypothesis). We hypothesized that the same immunomodulatory effect can have an impact on metabolic diseases like obesity, diabetes, hypertension and atherosclerosis, wherein inflammation plays a crucial role (extended hygiene hypothesis). To test this hypothesis, we examined the prevalence of lymphatic filariasis (LF) among diabetic, pre-diabetic and non-diabetic subjects who were part of the CURES (**C**hennai **U**rban **R**ural **E**pidemiology **S**tudy) study. In accordance with our hypothesis, we found reduced prevalence of LF among diabetic subjects compared to non-diabetic and pre-diabetic subjects. This was associated with decreased filarial antigen load and anti-filarial antibody levels. The association remained significant even after adjusting for socioeconomic status, age and gender. Interestingly, within the diabetic subjects, those who were filarial positive had reduced levels of pro-inflammatory markers (TNF-α, IL-6 and GM-CSF) compared to those who were filarial negative. In light of these findings, the decreasing incidence of filarial infection due to mass drug administration could potentially have an unexpected adverse impact on the prevalence of diabetes in India.

## Introduction

Global epidemiological studies have shown a marked increase in the incidence of diabetes worldwide. India leads the world in absolute numbers of diabetic subjects [1]. Type-2 diabetes mellitus constitutes about ∼90% of the entire diabetic population. The association between diabetes mellitus and increased susceptibility to infections is well known. Many diseases such as tuberculosis and candidiasis are more common in diabetic patients, while some such as invasive otitis externa and rhinocerebral mucomycosis occur almost exclusively in people with diabetes [2]. In addition, infections with group B streptococcus and Klebsiella spp. occur with increased severity in patients with diabetes and may be associated with an increased risk of complications [2].

Infection with systemic helminths, in addition to causing morbidity by themselves, may contribute to increased morbidity due to diabetes. But, there is very little data available on the prevalence of lymphatic filariasis (LF) among people with diabetes, although studies have examined the coexistence of LF with HIV [3], malaria [4] and tuberculosis [5]. Current estimates suggest that 129 million persons worldwide are infected with one of the three lymph-dwelling filariae (*Wuchereria bancrofti*, *Brugia malayi* or *B. timori*), the major causative agents of LF. The disease burden from LF is concentrated in tropical and sub-tropical countries (such as India) where the prevalence of type-2 diabetes is greatest [6]. This is particularly true in South India where the prevalence of LF caused by *W. bancrofti* is between 6–20% based on circulating filarial antigenemia [5]. Thus, in the present study, the influence of LF on diabetes was examined as part of an ongoing, prospective epidemiological study in Chennai, Southern India.

## Methods

### Study subjects

Institutional ethical committee approval from the Madras Diabetes Research Foundation Ethics Committee was obtained (Ref No-MDRF-EC/SOC/2009//05) and written informed consent was obtained from all the study subjects. Study subjects were recruited from the Chennai Urban Rural Epidemiology Study (CURES), an ongoing epidemiological study conducted on a representative population of Chennai (formerly Madras), the fourth largest city in India. The methodology of the study and the prevalence of diabetes in Chennai have been published elsewhere [7], [8]. Briefly, in Phase 1 of the urban component of CURES, 26,001 individuals were recruited based on a systematic sampling technique with random start. Fasting capillary blood glucose was determined using the OneTouch Basic glucometer (Lifescan, Johnson & Johnson, Milpitas, CA) in all subjects. Details of the sampling are described on our website (http://www.drmohansdiabetes.com/bio/WORLD/pages/pages/chennai.html). In Phase 2, detailed studies of diabetic complications, including nephropathy and retinopathy, were performed, and in Phase 3, every 10^th^ individual in Phase 1 was invited to participate in more detailed studies. As part of the questionnaire, the socio-economic details of the study participants was collected and recorded. For the present study, the following groups were randomly selected from Phase 3 of CURES, Group 1- 943 normal glucose tolerance subjects (NGT); Group 2- 154 subjects with impaired glucose tolerance (IGT); Group 3- 158 newly diagnosed type-2 diabetes subjects (ND-DM) and Group 4- 161 known type-2 diabetes subjects under treatment (KDM). A larger NGT group was included based on sample size calculations and to obtain baseline values for the normal population. The filarial status of these individuals was not known at the time of recruitment into the study.

### Anthropometric and biochemical parameters

Anthropometric measurements, including height, weight, and waist circumference, were obtained using standardized techniques. The body mass index (BMI) was calculated as the weight in kilograms divided by the square of height in meters. Fasting plasma glucose (FPG) (glucose oxidase-peroxidase method), serum cholesterol (cholesterol oxidase-peroxidase- amidopyrine method), serum triglycerides (glycerol phosphate oxidase-peroxidase-amidopyrine method), high density lipoprotein cholesterol (HDL-C) (direct method-polyethylene glycol-pretreated enzymes), and creatinine (Jaffe's method) were measured using a Hitachi-912 Autoanalyser (Hitachi, Mannheim, Germany). The intra- and inter assay coefficient of variation for the biochemical assays ranged between 3.1% and 5.6%. Glycated hemoglobin (HbA1c) was estimated by high pressure liquid chromatography using a variant machine (Bio-Rad, Hercules, CA). The intra- and inter-assay coefficient of variation of HbA1c was less than 5%.

### Detection of bancroftian LF

To quantify the filarial antigen levels and prevalence, sera were analyzed using the *W. bancrofti* Og4C3 antigen-capture enzyme-linked immunosorbent assay (Tropbio, James Cook University, Townsville, Queensland, Australia) according to the manufacturer's instructions.

### Determination of anti-filarial antibody titer

The serum antibody (IgG and IgG4) titer against *Brugia malayi* antigen (BmA) was determined by ELISA as described previously [9].

### Determination of serum cytokine levels

The levels of cytokines (TNF-α, IL-6, IL-1β, GM-CSF, IFN-γ, IL-13 and IL-10) in the undiluted serum were measured using a Bioplex multiplex cytokine assay system (Biorad, Hercules, CA). The lowest detection limit for the various cytokines were IL-1β -2.7pg/ml, IL-2-1.16 pg/ml, IL-4-0.3 pg/ml, IL-5- 2.08 pg/ml, IL-6- 2.31 pg/ml, IL-10- 2.2 pg/ml, IL-12- 2.78 pg/ml, IL-13- 2.22 pg/ml, IL-17 2.57, GM-CSF- 0.67 pg/ml, IFN-γ- 2.14 pg/ml and TNF-a- 4.89 pg/ml. TGF-β was estimated by conventional ELISA following manufacturer's protocol (R&D, Minneapolis, MN). The lowest detection limit for TGF-β was 7.8 pg/ml.

### Statistical analysis

All statistical analyses were performed using SPSS software (Version 15.0.0, Chicago).The prevalence of filarial infections among the different groups was analyzed by Pearson's Chi-Square test. The antigenic load and antibody titers were analyzed by Mann Whitney U test. The clinical and biochemical characteristics of the study subjects were compared using one-way ANOVA analysis. To account for multiple comparisons, the cytokine levels in controls (DM^−^LF^−^), DM^+^LF^+^ and DM^+^LF^−^ groups were compared by multinomial logistic regression analysis. P values<than 0.05 were considered significant.

## Results

### Study population characteristics

The baseline characteristics including demographics, clinical and biochemical features of the study population are shown in Table 1. As can be seen in the table, compared to subjects with normal glucose tolerance (NGT), those with glucose intolerance (i.e. IGT, ND-DM, KDM) had higher BMIs, systolic and diastolic blood pressure, serum cholesterol, LDL and triglycerides levels but lower HDL cholesterol levels. Since, socio-economic differences could be a confounding factor for the prevalence of both diabetes [10] and LF [11], the average monthly income of study subjects was recorded (Table 2). We also examined the occupation profile of the study subjects (Table S1). As can be seen, there were no significant differences in the socio-economic status between the various groups.

**Table 1:** Clinical and biochemical characteristics of study subjects.

PARAMETERS	NGT (n = 992)	IGT (n = 154)	ND-DM (n = 157)	KD (n = 160)	p value
**Age (yrs)**	38.1±13.2	47.2±14.3	45.6±11.3	50.6±13.7	**<0.001**
**Sex (M/F) (%)**	41.9/58.1	44.8/55.2	43.9/56.1	33.8/66.3	0.206
**BMI (Kg/m^2^)**	22.7±4.3	24.9±4.6	25.7±4.4	25.4±4.1	**<0.001**
**Duration of diabetes**	-	-	-	5.3±4.5	**-**
**Systolic blood pressure (mmHg)**	116.2±18.2	128.7±19.7	129.2±18.0	127.4±21.7	**<0.001**
**Diastolic blood pressure (mmHg)**	72.4±11.5	77.7±11.1	79.4±10.8	75.4±12.2	**<0.001**
**Fasting plasma glucose (mg/dl)**	83.9±7.8	94.7±12.8	148.8±62.1	162.1±68.3	**<0.001**
**Glycated hemoglobin (%)**	5.5±0.5	5.9±0.7	8.1±2.0	8.8±2.3	**<0.001**
**Total cholesterol (mg/dl)**	174.2±37.2	187.4±36.2	198.5±37.5	201.8±39.0	**<0.001**
**Serum triglycerides (mg/dl)**	109.8±62.5	150.3±113.9	184.8±140.7	180.2±130.6	**<0.001**
**High density lipoprotein cholesterol (mg/dl)**	43.2±9.8	42.4±12.2	40.5±7.4	41.6±9.3	**<0.01**
**Low density lipoprotein cholesterol (mg/dL)**	109.1±32.0	115.0±34.1	121.0±34.3	124.1±39.0	**<0.001**
**Creatinine (mg/dL)**	0.88±0.38	0.881±	0.87±.18	0.75±.19	**<0.001**
**Microalbuminuria (mg/dL)**	13.6±29.3	18.7±32.9	20.2±26.0	37.3±57.0	**<0.001**

**Table 2:** Socioeconomic status of study subjects*.

Monthly Income (Rs.)	NGT %	IGT %	ND-DM %	KDM %	LF+ %	LF− %
<2000	32.9	33.3	25.2	39.7	33	32.5
2000–5000	50.1	46.1	54.8	51.6	49	50.4
5000–10000	14.5	15.6	17.0	4.8	16	13.8
10000–20000	2.0	3.5	2.2	3.2	2	2.6
>20000	0.5	1.4	0.7	0.8	NIL	0.7

*The occupation of the study subjects were collected as part of the CURES questionnaire.

### Prevalence of LF

**Figure 1:**
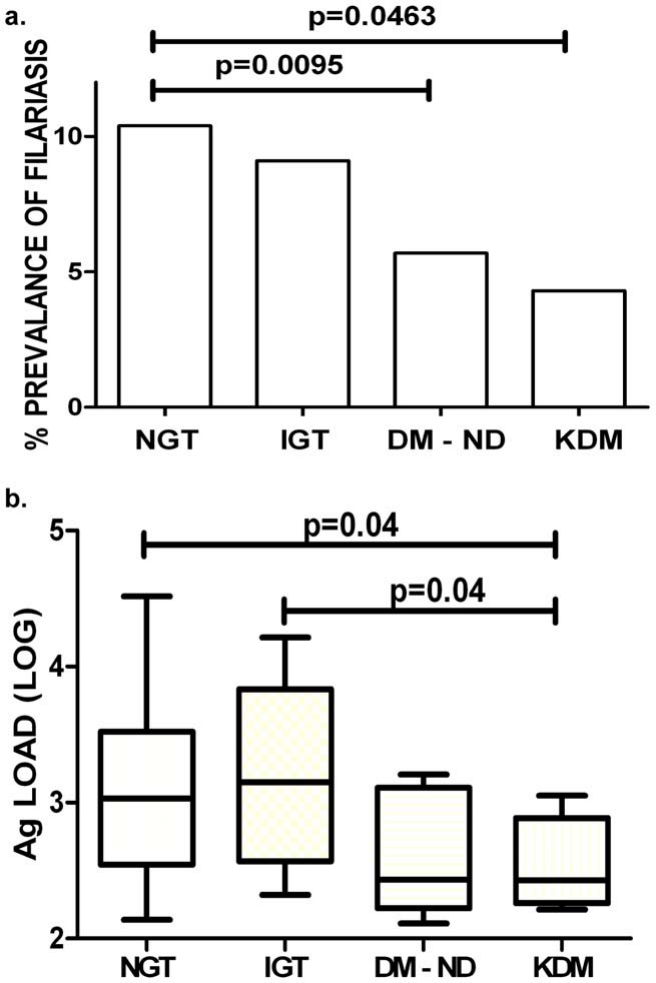
Reduced prevalence of Lymphatic Filariasis (LF) among diabetic individuals. Prevalence of filarial infection in diabetic (DM-ND-Newly diagnosed and KDM-Known diabetic) and non-diabetic (NGT-Normal glucose tolerance and IGT-Impaired glucose tolerance) subjects was estimated (Panel a). The percentage of LF positive individuals in each group is shown. Statistical difference in the prevalence was determined by chi-square analysis. Filarial antigen levels in diabetic (DM-ND-Newly diagnosed and KDM-Known diabetic) and non-diabetic (NGT-Normal glucose tolerance and IGT-Impaired glucose tolerance) subjects with active filarial infection (Panel b). Statistical differences in the antigenic load were determined by Mann-Whitney U test.

The prevalence of LF among the various groups was determined by quantitative TropBio ELISA (>128 U/ml) and differences in the prevalence of LF among the groups were observed. The prevalence of LF was found to be 10.4% in the NGT, 9.1% in IGT, 5.7% in ND-DM and 4.3% in KDM respectively. The differences in the prevalence rate between NGT and KDM (p = 0.0463) and NGT and ND-DM (p = 0.0095) were significant (Fig 1a).

### Decreased circulating filarial antigen levels and decreased anti-filarial antibody titer among type-2 diabetes subjects

To examine more quantitatively the association of type-2 diabetes and LF, we next quantified the serum circulating filarial antigen (CFA) levels among the filarial positive subjects. Not only was there a difference in prevalence rates between those with NGT and those with glucose intolerance but there was also a clear difference in the absolute levels of CFA in the LF-infected individuals, with CFA levels being lower among the diabetic groups compared to the NGT group (Fig 1b). The geometric mean (range) of CFA levels in the four groups were: NGT-1,594 (127–32,768), IGT-1,520 (209–16,345), ND-DM-929 (129–32,768) and KDM-351 (163–1,126) with the differences in the antigen levels between the KDM and the NGT (p = 0.04) and the KDM and the IGT (p = 0.04) being statistically significant.

**Figure 2:**
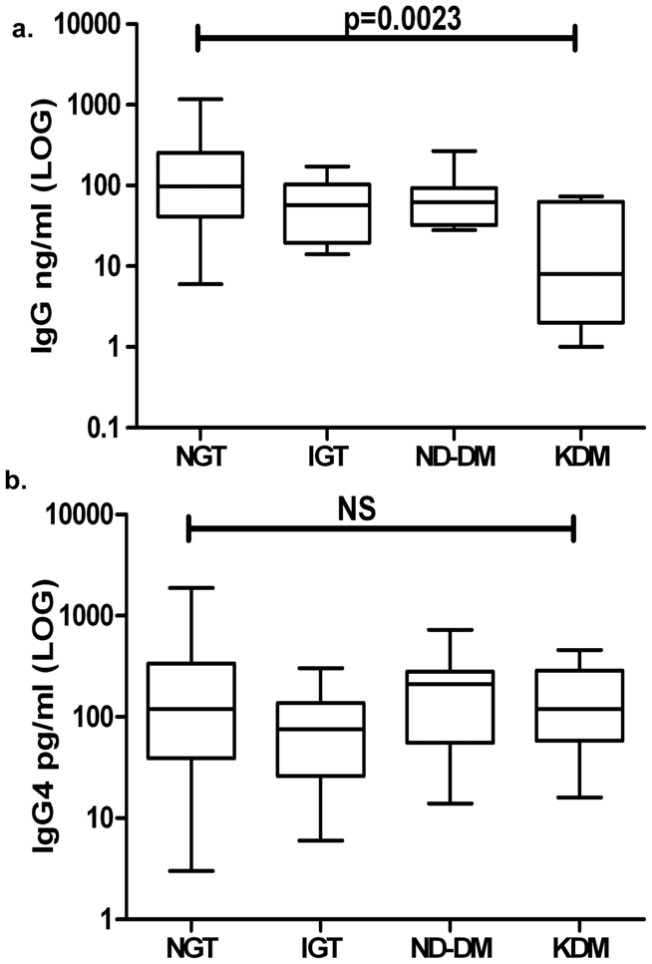
Anti-filarial antibody levels in diabetic (DM-ND-Newly diagnosed and KDM-Known diabetic) and non-diabetic (NGT-Normal glucose tolerance and IGT-Impaired glucose tolerance) subjects with active filarial infection. Box and whisker plots of the log transformed levels of IgG (Panel a) and IgG4 (Panel b) are shown for each of the four groups. NS = Not significant. Statistical differences in the antibody titer were determined by Mann-Whitney U test.

We next quantified the serum anti-filarial antibody levels among those with LF in the four groups (Fig 2). Even though the mean IgG4 levels were not different among the four groups, the mean IgG levels were significantly lower in the KDM compared to NGT (p<0.0023),

### Differential serum cytokine profile in subjects with type-2 diabetes and/or LF

**Figure 3:**
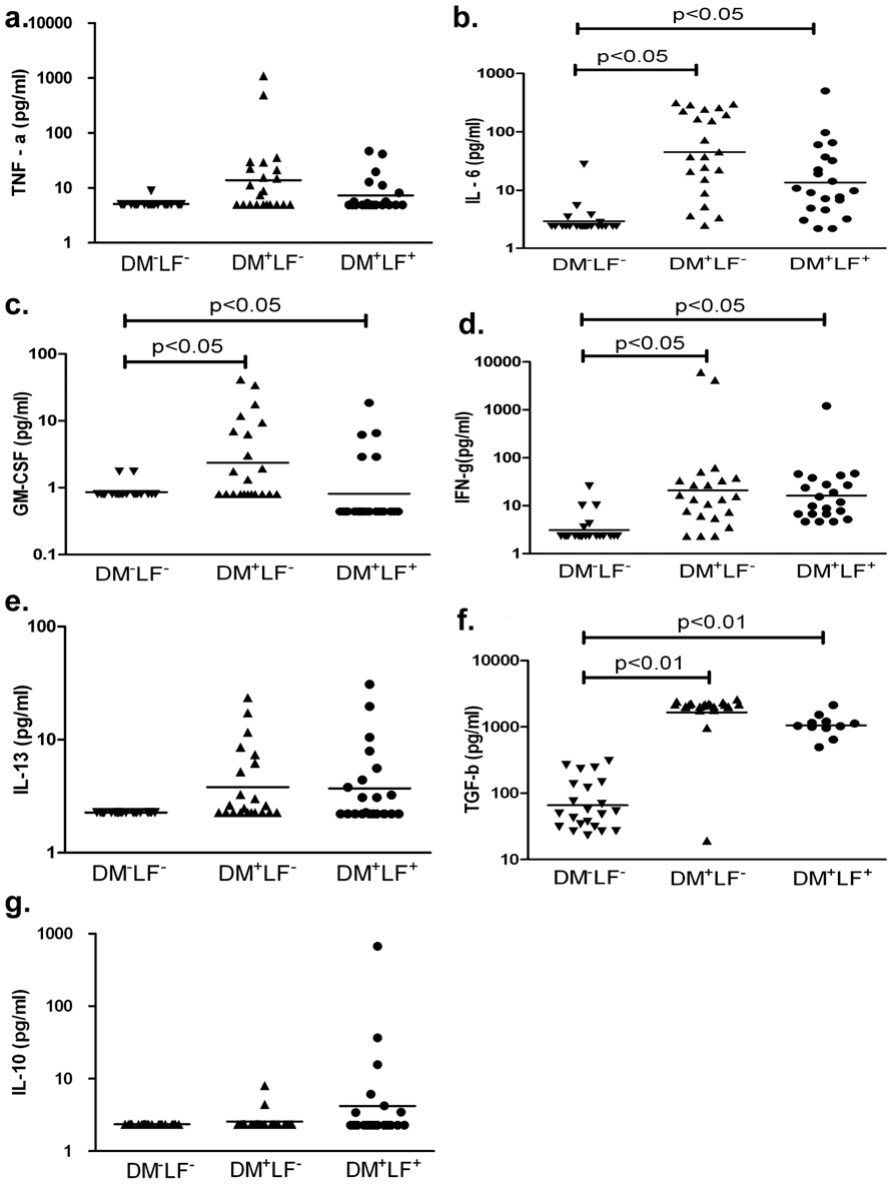
Pro- and anti - inflammatory serum cytokine levels in control (DM^−^LF^−^), diabetes alone (DM^+^LF^−^) and diabetes and LF (DM^+^LF^+^) subjects. Serum levels of TNF-α (Panel a), IL-6 (Panel b), GM-CSF (Panel c), IFN-γ (Panel d), IL-13 (Panel e), TGF-β (Panel f) and IL-10 (Panel g) are shown. Each dot represents an individual patient, with the geometric mean represented by the horizontal bars. p values were calculated by multinomial logistic regression analysis.

We next quantified pro- and anti-inflammatory cytokine levels among control (DM^−^LF^−^), diabetic only (DM^+^LF^−^) or both LF and diabetic (DM^+^LF^+^) subjects (Fig 3). In comparison to controls, the diabetes only (DM^+^LF^−^) group had high levels of IL-6 and GM-CSF, which was significant (p<0.01) (Fig 3b and c). There was no significant difference in the levels of TNF- α (Fig 3a). In DM^+^LF^+^ subjects, there was a significant reduction in the levels of IL-6 and GM-CSF compared to the diabetic only group (DM^+^LF^−^) (geometric mean (GM) of 13.57 pg/ml versus 45.13 pg/ml for IL-6, p<0.05; and GM of 0.81 pg/ml versus 2.24 pg/ml for GM-CSF, p<0.05). TNF- α levels were not significantly different between the two groups. IL-1β levels were not statistically different among the three groups (data not shown). IFN-γ levels were significantly elevated in diabetic only group compared to controls but was unaltered by the LF status (Fig 3d).

When the levels of anti-inflammatory cytokines were measured, both the diabetic groups (DM^−^LF^+^ and DM^+^LF^+^) had comparable levels of IL-13 and TGF-β (Fig 3e and f), but TGF-β was significantly higher in both the diabetic groups compared to controls (p<0.01). No significant difference was seen in IL-10 levels among the three groups (Fig 3g).

## Discussion

It is well known that individuals with diabetes are at increased risk of susceptibility to several infectious diseases including tuberculosis [12], urinary tract infection [13] and mucormycosis [14]. It is generally assumed that diabetes increases the susceptibility to all infections [2]. But in this study, we clearly demonstrate that the prevalence of LF is lower in subjects with type-2 diabetes. A serial decline in the prevalence of LF was seen as individuals progressed from NGT to IGT to ND-DM to KDM with the significance being maintained even after adjusting for age and gender. The decrease in prevalence was associated with decreased antigen load and anti-filarial IgG antibody titer but the anti-filarial IgG4 titer was unaffected. The reduced IgG levels in diabetic subjects was expected since previous reports have shown reduced levels of IgG and increased levels of IgA among diabetic subjects [15], [16]. In terms of humoral responses, both subjects with active filarial infection and those who are exposed but resistant to infection mount vigorous antibody responses to parasite antigen, most specifically, IgG4 [9], [17]. Thus, BmA- specific IgG4 can be considered to be a good surrogate marker for exposure. The fact that IgG4 levels were not significantly different between the four groups, is further evidence to show that, differences in exposure to infection is less likely a reason for the differences in prevalence between the diabetic and non-diabetic groups. It is very unlikely that the decreased prevalence of LF among diabetic subjects was due to LF-mediated mortality, as LF is a chronic, non-lethal disease. Since, differences in socio-economic status could be a confounding factor; the average monthly income and occupation of the study subjects were analyzed. No significant difference was seen between the study groups, indicating that, the socio-economic status is unlikely to be a potential confounding factor for the differences seen in the prevalence of LF. Another confounding factor that could be of significance is the nutritional differences [18] that could arise due to calorie restriction among diabetic subjects. But, there was no difference with respect to average protein intake (approximately 12%) among different groups, again suggesting that nutritional differences or dietary intake are unlikely to be potential confounding variables in the study. More likely is the interplay between two chronic conditions in which the longstanding regulatory environment seen in LF may play a role in conferring resistance to type-2 diabetes. There are some reports that have documented a similar inverse relationship between diabetes and hepatitis C infection [19], but other studies failed to reproduce the association [20]. More studies are thus needed on the coincidence of diabetes and other infectious diseases to have a better understanding about the interplay of infection/inflammation and diabetes.

In mice, there is evidence to show that filarial infection can prevent type-1 diabetes (“hygiene hypothesis”) [21], but whether the same immunomodulatory effect can dampen inflammation and protect against type-2 diabetes is currently not known. To better understand the mechanism associated with the decreased prevalence of LF among diabetic subjects, we studied the serum cytokine levels. The main focus was to determine the effect of LF on diabetes in terms of the serum cytokine profile. Although filarial parasites elicit a broad spectrum of inflammatory and regulatory responses mediated by cytokines, whether this type of immunomodulation occurs in co-incident diabetes has not been well-studied. The serum cytokine profile of LF only patients has already been reported by Satapathy et al. [22] and us, previously [23]. Diabetes in conjunction with LF had decreased levels of TNF-α and IL-6 (cytokines that have already been associated with insulin resistance (IR) [24]), compared to those with DM alone. Diabetic subjects (without LF) had a typical pro-inflammatory phenotype with high levels of TNF-α, IL-6 and GM-CSF. IL-1β, however, was not elevated in these subjects, although it has been previously been shown to act synergistically with TNF-α and IL-6 in inducing IR [24]. The contribution of GM-CSF (seen to be elevated in diabetic subjects) in mediating IR is currently not known. Interestingly, in those with both diabetes and LF, the pro-inflammatory cytokines - TNF-α, IL-6 and GM-CSF were reduced, compared to those without LF, suggesting that LF-mediated reduction of pro-inflammatory cytokines negatively influences the development of IR.

Although pro-inflammatory cytokine levels are typically elevated in diabetic subjects, the data on the balance of effector T cell phenotypes in this condition has been confusing with some studies reporting Th1 polarization [25], others reporting Th2 skewing [26], and still others reporting a balanced response [27]. In the present study, diabetic subjects (who were LF negative) had very high levels of IFN-γ and IL-13 suggestive of a mixed (relatively non-polarized) phenotype. Whether this immune phenotype is the cause or effect of IR remains to be established.

Although the anti-inflammatory cytokines - IL-10 and TGF-β - have largely been associated with immune-regulation in LF (with IL-10 playing the major role), the down regulation of pro-inflammatory cytokines in type-2 diabetic subjects with LF seems to be due to TGF-β and not IL-10. However, as TGF-β is often elevated in diabetic subjects, other immunomodulatory molecules such as CTLA-4, PD-1, IDO might act in concert with TGF-β in the modulation of the immune responses in these groups [28].

Our study suggests that one mechanism by which LF can potentially protect against type-2 diabetes, is by modulating the pro-inflammatory environment seen in diabetes. Helminth infections, such as LF could modulate diabetes by inducing a chronic, non-specific, low-grade, immune regulation mediated by Th2/Tregs (modified Th2 response) which in turn can suppress the pro-inflammatory responses [29]. One can speculate that, childhood filarial infection reduces TNF-α, IL-6 and GM-CSF levels thereby conferring protection against type-2 diabetes. An alternate hypothesis could be that the inflammation associated with diabetes may also promote anti-filarial immunity by enhancing anti-filarial antibody responses that could mediate parasite clearance [30].

Although this study suffers from the limitation of being a cross-sectional study and therefore not providing a direct causative mechanism for the decreased prevalence of LF among diabetic subjects, the study highlights the importance of the need to understand the complex interactions between an infectious disease (LF) and a metabolic disorder (Type-2 diabetes). In addition, the immune as well as non-immune mechanisms by which the interplay between LF and DM occurs needs to be explored in detail. Finally, the decreasing incidence of LF (due to mass eradication programs) could have an impact on the incidence of type-2 diabetes in the future and obviously this is an important area for future study.

## Supporting Information

Table S1 The occupation of the study subjects were collected as part of the CURES questionnaire.
(0.05 MB DOC)
