# Supplementary material for: Decreased Prevalence of Lymphatic Filariasis among Diabetic Subjects Associated with a Diminished Pro-Inflammatory Cytokine Response (CURES 83)
Source: PLoS Negl Trop Dis. 2010 Jun 15;4(6):e707. doi: 10.1371/journal.pntd.0000707 (PMC2886036; doi:10.1371/journal.pntd.0000707)
Supplement: Table S1 — The occupation of the study subjects were collected as part of the CURES questionnaire. (0.05 MB DOC) [file pntd.0000707.s001.doc]

**TABLE- S1**

| **Grade** | **NGT (%)** | **IGT (%)** | **NDDM (%)** | **KDM (%)** |
| --- | --- | --- | --- | --- |
| **1** | 34.1 | 24.8 | 34.1 | 31.7 |
| **2** | 3.4 | 0 | 5.9 | 0 |
| **3** | 7.8 | 4.3 | 0 | 8.2 |
| **4** | 0 | 0 | 0 | 0 |
| **5** | 0.6 | 0 | 0 | 0 |
| **6** | 3.6 | 8.7 | 8.8 | 5.3 |
| **7** | 25.9 | 43.5 | 26.5 | 33.4 |
| **8** | 0 | 0 | 0 | 0 |
| **9** | 24.6 | 18.7 | 24.7 | 21.4 |

**OCCUPATION OF STUDY SUBJECTS***

1. Professional/ Executive/Manager/Big business
2. Clerical /Medium business
3. Sales
4. Agriculture / self employed
5. Household & domestic
6. Services
7. Skilled manual
8. Unskilled manual
9. Do not work /Unemployed

* The occupation of the study subjects were collected as part of the CURES questionnaire

**Occupation (Category)**

**1.Professional/ Executive/Manager / Big business**

**1.** Physical scientists

**2.** Architects, engineers, technologists and surveyors

**3.** Engineering technicians

**4.** Aircraft and ships officers

**5.** Life scientists

**6.** Life science technicians

**7.** Physicians and surgeons

**8.** Nursing and other medical and health technicians

**9.** Scientific, medical and technical persons, other

**10.** Mathematicians, statisticians and related workers

**11.** Economists, and related workers

**12.** Accountants, auditors and related workers

**13.** Social scientists and related workers

**14.** Jurists

**15.** Teachers

**16.** Poets, authors, journalists and related workers

**17.** Sculptors, painters, photographers, other creative artists

**18.** Composers and performing artists

**19.** Professional workers

**20.** Elected and legislative officials

**21.** Administrators, officials government and local bodies

**22.** Proprietors, directors and managers, wholesale, retail trade

**23.** Directors and managers, financial institutions

**24.** Proprietors, directors, manufacturing managers and related

**25.** Proprietors, directors, managers, transport, communication

**26.** Working proprietors, directors and managers, other services

**27.** Administrative, executive and managerial workers

**28.** Computing machine operators

**29.** Transport and communication supervisors

**30.** Manufacturers, agents

**31.** Hotel and restaurant keepers

**2. Clerical /Medium business**

**1.** Clerical and other supervisors

**2.** Stenographers, typist and card and tape punching operators

**3.** Book keepers, cashiers and related workers

**4.** Clerical and related workers

**3. Sales**

**1.** Merchants and shopkeepers, wholesale and retail trade

**2.** Technical salesmen and commercial travellers

**3.** Salesmen, shop assistants and related workers

**4.** Insurance, real estate, securities, salesmen, auctioneers

**5.** Money lenders and pawn brokers

**6.** Sales workers

**4.Agriculture / self employed**

**1.** Farm plantation, dairy and other managers and supervisors

**2.** Cultivators

**3.** Farmers other than cultivators

**4.** Agricultural labourers

**5.** Plantation labourers and related workers

**6.** Other farm workers

**7.** Forestry workers

**8.** Hunters and related workers

**9.** Fishermen and related workers

**5.Household & domestic**

**1.** House keepers, matrons and stewards

**2.** Maids and other house keeping service workers

**6.Services**

**1.** Village officials

**2.** Transport conductors and guards

**3.** Mail distributors and related workers

**4.** Telephone and telegraph operators

**5.** Cooks, waiters, bartenders and related workers

**6.** Building caretakers, sweepers, cleaners and related workers

**7.** Protective service workers

**8.** Service workers

**7. Skilled manual**

**1.** Hair dresser, barbers, beauticians and related workers

**2.** Miners, quarrymen, well drillers and related workers

**3.** Metal processors

**4.** Wood preparation workers and paper makers

**5.** Chemical processors and related workers

**6.** Spinners, weavers, knitters, dyers and related workers

**7.** Tanners, fellmonger and pelt dressers

**8.** Food and beverage processors

**9.** Tobacco prepares and tobacco product makers

**10.** Tailors, dress makers, sewers, upholsterers and related

**11.** Shoemakers and leather good makers

**12.** Carpenters, cabinet and related workers

**13.** Stone cutter and carvers

**14.** Blacksmiths, tool makers and machine tool operators

**15.** Machinery fitters, assemblers, precision instrument makers

**16.** Electrical fitters and related electrical electronic workers

**17.** Broadcasting and sound equipment operators, projectionists

**18.** Plumbers, welders, sheet metal and structural metal

**19.** Jewellery and precious metal workers and metal engravers

**20.** Glass formers, potters and related workers

**21.** Rubber and plastic products makers

**22.** Paper products makers, printing and related workers

**23.** Painters, production and other construction workers

**24.** Stationery engines and related equipment operators

**25.** Transport equipment operators

**8. Unskilled manual**

**1.** Launderers, dry- cleaners and pressers

**2.** Labourers

**9. Do not work /Unemployed**

None including housewives, students, unemployed, and retire
